# Effects of Oxygen Element and Oxygen-Containing Functional Groups on Surface Wettability of Coal Dust with Various Metamorphic Degrees Based on XPS Experiment

**DOI:** 10.1155/2015/467242

**Published:** 2015-07-15

**Authors:** Gang Zhou, Cuicui Xu, Weimin Cheng, Qi Zhang, Wen Nie

**Affiliations:** ^1^State Key Laboratory of Mining Disaster Prevention and Control Co-Founded by Shandong Province and the Ministry of Science and Technology, Shandong University of Science and Technology, Qingdao 266590, China; ^2^College of Mining and Safety Engineering, Shandong University of Science and Technology, Qingdao 266590, China

## Abstract

To investigate the difference of surface oxygen element and oxygen-containing functional groups among coal dusts with different metamorphic degrees and their influence on surface wettability, a series of X-ray photoelectron spectroscopy experiments on 6 coal samples are carried out. The result demonstrates that the O/C ratio of coal surface shows an overall increasing trend compared with the result of its elements analysis. As the metamorphic degree increases, the O/C ratio on the surface gradually declines and the hydrophilic groups tend to fall off from coal surface. It could be found that different coals show different surface distributions of carboxyl and hydroxyl which are considered as the greatest promoter to the wettability of coal surface. With the change of metamorphic degree, the distribution of ether group is irregular while the carbonyl distribution keeps stable. In general, as the metamorphic degree goes higher, the content of oxygen-containing polar group tends to reduce. According to the measurement results, the contact angle is negatively related to the content of oxygen element, surface oxygen, and polar groups. In addition, compared with surface oxygen content, the content of oxygen-containing polar group serves as a more reasonable indicator of coal dust wettability.

## 1. Introduction

As a serious threat to safety of mine production and health of miners, coal dust is deemed to be one of the major hazards in coal production process. On the one hand, explosions by coal dust will cause severe accidents; on the other hand, miners exposed to the environment directly, which was contaminated by coal dust, are highly susceptible to pneumoconiosis. From early 2000 to the end of 2014, 480 mine workers died from 14 explosions caused by coal dust. Since the 1950s, more than 727000 cases of pneumoconiosis and 150000 deaths in coal mine have been reported. In 2013 alone, 15079 pneumoconiosis cases are recorded with respect to mine production in China. The total medical expenses for the treatment of pneumoconiosis are as high as 8 billion RMB annually in China [1–3]. At present, the most widely used means of coal production in China is still wet dust removal method, which included coal seam water injection and dust suppression by spraying and dedusting fan. According to analyzed results, the maximum concentration of dust after wet dust removal is nearly 1500 mg/m^3^ in fully mechanized mining face and is close to 500 mg/m^3^ in fully mechanized driving face [4, 5]. The main reason is that most of the coal dust in China has poor wettability, which leads to a limited effect of the wet dust removal method applied to underground work place. Therefore, it is important to study wet dedusting to achieve higher dust removal efficiency on the fully mechanized face. For a long time most of the studies on coal dust wettability are focused on the macroscale content, such as contact angle and settlement test [6]. Few researchers have analyzed the mechanism of coal dust wettability from microscopic point of view.

Wettability can be judged by measuring the contact angle of liquid and dust [7, 8]. On the basis of that, the surface of coal consists of three kinds of states: strongly hydrophobic, weakly hydrophobic, and hydrophilic [9]. Surfactants, especially multivalent metal ions, could improve the wettability of coal dust by reducing the surface tension of liquids effectively [10, 11]. Glanville et al. showed that the rate of surface wetting was influence by surfactant concentration, temperature, granularity of coal dust, and area of the wetting surface through the Walker Test [12–14]. Osasere Orumwense has studied the effect of electrolyte coagulants and flocculants on coal surface properties at various amounts of pH values. His results showed that the adsorption of flocculants similar to surfactants occurred through physical interaction with the coal surface, possibly due to hydrophobic bonding [15]. Since then, scholars studied the different wettability of organic liquids on the surface of coal dust. It showed that the wettability of the coal increased with the increase of the polarity of the liquid [16]. Wetting agents are useful in some coal mines significantly but had no effect on certain mines. This is mainly because the characters of dust have a great effect on wettability of coal dust, which has a great influence on dust suppression effect. Therefore, without the characters of dust, we will never acquire a better dust-proof effect. By exploring the relationship between components of coal dust and the contact angle, mineral matter [17, 18], state of surface oxidation [19], fractal dimension [20], and so forth have an impact on the wettability (contact angle). Few researchers have analyzed the mechanism of coal dust wettability in microscopic perspective. With the development of modern technologies, the emergence of high-precision test instruments has made it possible to analyze the microscopic molecular structure of coal dust [21]. X-ray photoelectron spectroscopy (i.e., XPS), which was widely used in chemical structure analysis for its ability to efficiently observe elemental composition and functional group information on coal surface within 10 nm, is one of the most effective methods for element investigation.

The oxygen-containing functional group is vital to coal surface properties, such as hydrophilicity and hydrophobicity. The investigation on the relation between coal dust wettability and its oxygen-containing functional group is helpful to uncover the characters of wettability from a microscopic view. Therefore, in the present work, a series of X-ray photoelectron spectroscopy experiments have been carried out for different coals from a perspective of surface oxygen element and oxygen-containing groups. The differences among various types of coal dusts are obtained in terms of surface element and oxygen-containing functional group. The influence of surface element and oxygen-containing functional group on coal surface wettability is analyzed, and our results explain and demonstrate the wettability mechanism of coal dust.

## 2. Experimental Procedure

### 2.1. Proximate and Elemental Analysis of Coal Samples

Proximate analysis and elemental analysis reflect properties of coal from different aspects and both can serve as the reference on the features of coal quality. In the present work, 6 representative coal samples are selected as they are of various ranks and from different areas. Proximate analysis and elemental analysis are performed according to national standard GB/T212-2008 (proximate analysis of coal) and GB476-91 (elemental analysis of coal), and the results are shown in Table 1.

It can be noted in Table 1 that as the metamorphic degree goes higher, the most obvious changes in coal are the increase of carbon and decrease of oxygen. The levels of moisture, ash, and volatile content vary according to the type of coal, which means that the metamorphic environment plays a significant role. However, the results show no clear relevance between these parameters and the metamorphic degree.

### 2.2. XPS Experiment of Different Coal Samples

#### 2.2.1. Pretreatment of Coal Samples

The samples are crushed to a size below 80 meshes. To prevent the effect of inorganic ash on the characterization of the surface oxygen-containing groups, the samples are deashed. First, the samples are treated by a solution (the volume ratio of deionized water to 37% HCl and 40% HF is 2 : 1 : 1) in a 300 mL beaker for 6 h at 50°C. Then, it is washed with deionized water until the solution becomes neutral. After that, the sample is dried in a vacuum oven at 105°C for 24 h.

#### 2.2.2. XPS Full-Scan

XPS is a surface-sensitive and quantitative spectroscopic technique that is able to analyze chemical bonds on coal surface within 10 nm depth. Coal surface wettability indicates the feasibility of surface to contact water. Through XPS, the distribution of coal surface elements and groups can be obtained. The quantitative information of the main hydrophilic groups (i.e., surface oxygenic functional groups) can also be acquired to further investigate coal wettability from a microcosmic point of view.

**Figure 1:**
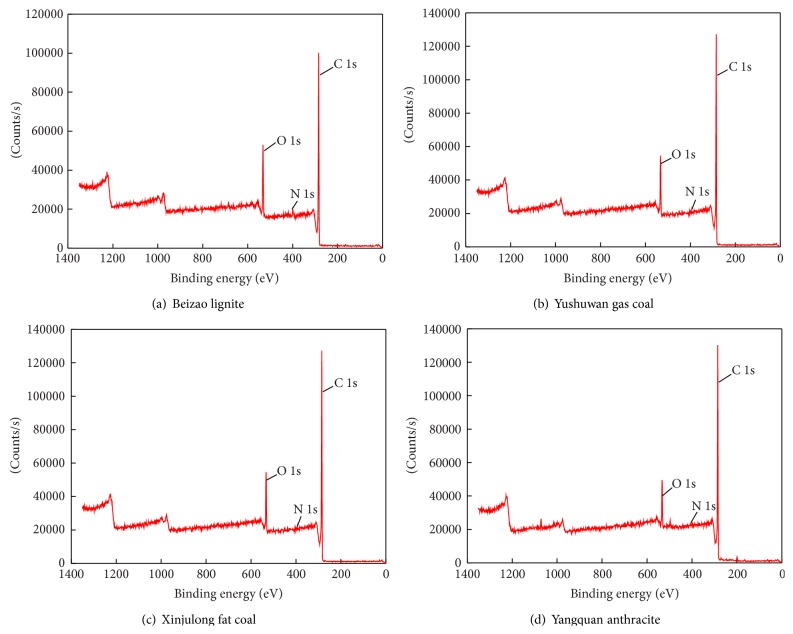
Chart of XPS full spectrum scan of coal samples with different metamorphic grades.

The test is performed by a multifunctional photoelectron spectrometer (Thermo Scientific ESCALAB250Xi) with Al K*α* X-ray. The equipment works under a power of about 200 W and a beam spot diameter of 500 *μ*m. The analysis is done under vacuum condition with a pressure of 3 × 10^−10 ^mbar. The binding energies are calibrated using the C1s (electrons in the 1s subshell) peak with a reference of 284.8 eV. In the XPS spectra, the ordinate represents the electrical counter, and abscissa stands for binding energy (BE). First, the XPS full-scan is performed to gain the element information of the coal surface. As the inorganic mineral has been almost removed, the peaks detected are mainly corresponding to organic materials. The full-scan XPS spectra of coals with different degrees of metamorphism (Beizao brown coal, Yushuwan gas coal, Xinjulong fat coal, and Yangquan anthracite) are shown in Figure 1.

## 3. Results and Discussion

### 3.1. Surface Oxygen Content of Coals with Different Degrees of Metamorphism

As displayed in Figure 1, the peak height reveals the content of elements, in which C and O take up the majority. The peaks corresponding to these two elements are obvious while no evident peak is associated with N and S due to their low content. The full-scan data of XPS are listed in Table 2.

The data in Table 2 demonstrate the following conclusions:

1. As the metamorphic degree increases, the carbon content on coal surface goes up and the oxygen content declines, which is consistent with the elemental analysis results.
2. Compared with the elemental analysis results, the O/C ratio on the surface of all the coal samples shows an increasing trend, indicating that oxygen element is widely distributed on coal surface rather than the whole volume of coal dust. The O/C ratio has the greatest increase on the surface of highly metamorphic coals (coking coal and anthracite) compared with the elemental analysis results.
3. For all the coal samples, the O/C ratio on coal surface decreases with an increasing metamorphic degree.

### 3.2. Content Analysis of Oxygen-Containing Functional Groups on Coal Surface

#### 3.2.1. Analysis of Oxygen-Containing Functional Groups

**Figure 2:**
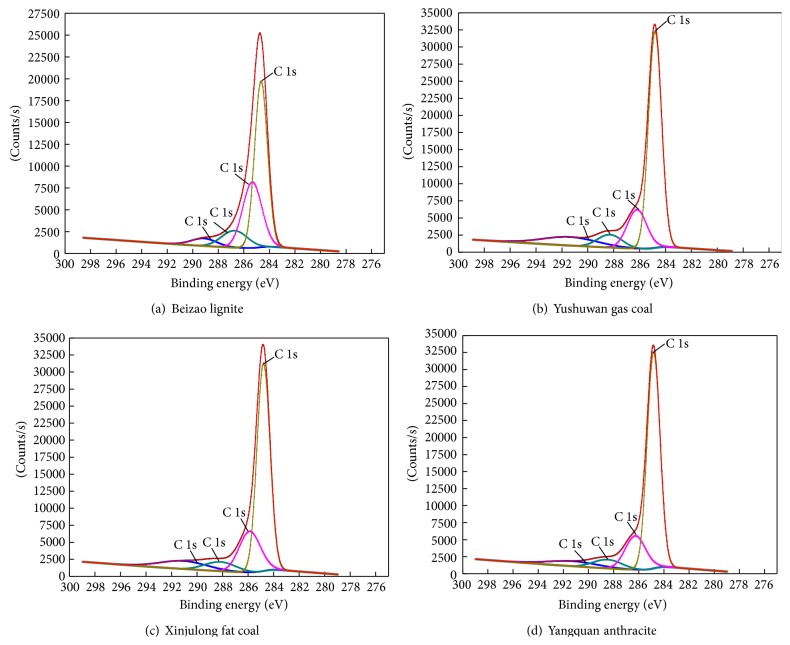
XPS peak chart of coal samples with different metamorphic grades.

The organic oxygen in coal is bound to carbon atom. The valence state of the adjacent oxygen can be detected from the chemical environment of carbon atom. Therefore, narrow scan of carbon is used to obtain the information of oxygen-containing functional groups. The XPS spectrogram at 1s is split and fitted by XPS PEAK software and the curves are plotted using Origin software. The peak-split graphs of coal samples with different metamorphic degrees (Beizao lignite, Yushuwan gas coal, Xinjulong fat coal, and Yangquan anthracite) are illustrated in Figure 2.

Through peak-split and fitting process, the carbon spectrogram can be divided into four peaks with different energy intensity: the peak at 284.6 eV indicates hydrocarbons (C–H, C–C); the characteristic peak at 286.2 eV relates to phenol or ether carbons (C–O); the characteristic peak at 288.1 eV denotes carbonyl carbons (C=O); and the peak at 289.9 eV is for carboxyl carbon (COO) [22]. The method of elemental sensitivity factor is employed to convert peak areas to element contents and obtain the relative content of carbon element with different forms. The classification and relative content of organic carbons in the six coal samples are shown in Table 3.

As seen in Table 3, the oxygen-containing functional groups in coal can be classified into three types: carbon-oxygen single bond (mainly ether (C–O–C) and hydroxyl (C–OH)), carbonyl group (C=O), and carboxyl group (COO). The oxygen in the carbonyl, carboxyl, and hydroxyl group may affect the chemical state of only one carbon atom in the functional groups, whereas the hydroxyl can affect the state of two atoms. The oxygen-containing functional groups of different coal samples have the same form but relatively different contents, leading to different wettability.

#### 3.2.2. Content Analysis of Surface Oxygen-Containing Functional Groups

The molar content percentage of surface oxygen-containing functional groups can be calculated based on the atom concentration in the full-scan spectrum of XPS. Meanwhile, the relative content of carboxyl and carbonyl groups can be obtained from their peak-split results. Limited by the measurement precision of XPS, there is no distinct difference between ether group and hydroxyl group. However, an equation concerning the oxygen in C–O bond can be formed from the peak-split result as both functional groups have an oxygen atom. In addition, another equation can be established from the conservation of carbon atom, which includes one hydroxyl carbon atom and two ether carbon atoms in C–O bond. From these two relations above, the relative molar content of carboxyl and carbonyl groups is calculated.

To illustrate the calculation process for the concentration of oxygen-containing functional groups in different chemical forms, a case of Beizao lignite is shown as follows and other coals have similar calculation process:

1. The relative molar concentration of carboxyl group: *W*_mol_(COO) = 9.22 *∗* 80.34% = 7.41%.
2. The relative molar concentration of carbonyl group: *W*_mol_(C=O) = 3.85 *∗* 80.34% = 3.09%.
3. The relative molar concentration of ether and hydroxyl group is as follows:
  (a) The molar ratio of C–O bond to the atoms in the oxygen-containing functional groups: *W*_mol_(C–O) = 20.37/(9.22 + 3.85 + 20.37) *∗* 100% = 60.92%.
  (b) The oxygen concentration of hydroxyl and ether group: *W*_mol_(O) = 60.92%  *∗* 18.22% = 11.10%.
  (c) The equation of the oxygen in C–O bond: *W*_mol_(–O–) + *W*_mol_(–OH) = 11.10%.
  (d) The equation concerning the carbon in C–O bond: *W*_mol_(2C_–O–_) + *W*_mol_(C_–OH_) = 80.34%  *∗* 20.37% = 16.37%.

Solving the two equations from (c) and (d), the molar concentrations are obtained: *W*_mol_(–O–) = 5.27% and *W*_mol_(–OH) = 5.83%.

The calculation results of surface oxygen-containing functional groups from all the six coal samples are tabulated in Table 4.

Among the oxygen-containing functional groups, carboxyl group is considered the greatest promoter to the wettability of coal surface, followed by hydroxyl group [23, 24]. By analyzing the data in Table 4, it could be found that various coals show different distributions of carboxyl and hydroxyl on surface. In general, as the metamorphic degree goes higher, the contents of surface carboxyl and hydroxyl tend to reduce while the content of carbonyl remains stable. The content of ether, however, seems to be irregularly fluctuating with the change of metamorphic degree. The oxygen-containing polar group is unevenly distributed on the surface of the coals and its content falls from 13.24% to 4.74% as the metamorphic degree increases.

## 4. The Influence of Oxygen (Group) on Coal Wettability before and after Being Deashed

Generally the qualitative methods for the determination of coal dust wettability include powder immersion speed method, water membrane flotation, and water vapor adsorption. As for the quantitative method, however, the surface wettability is usually determined by measuring the parameters such as the electric potential *ζ*, contact angle, and wetting heat. In our experiment, contact angle is introduced to characterize the macroscopic wettability. The optical instrument (DSA100) is used to measure the contact angle between coal dust and distilled water before and after the coal samples are deashed. The result is shown in Table 5.

**Figure 3:**
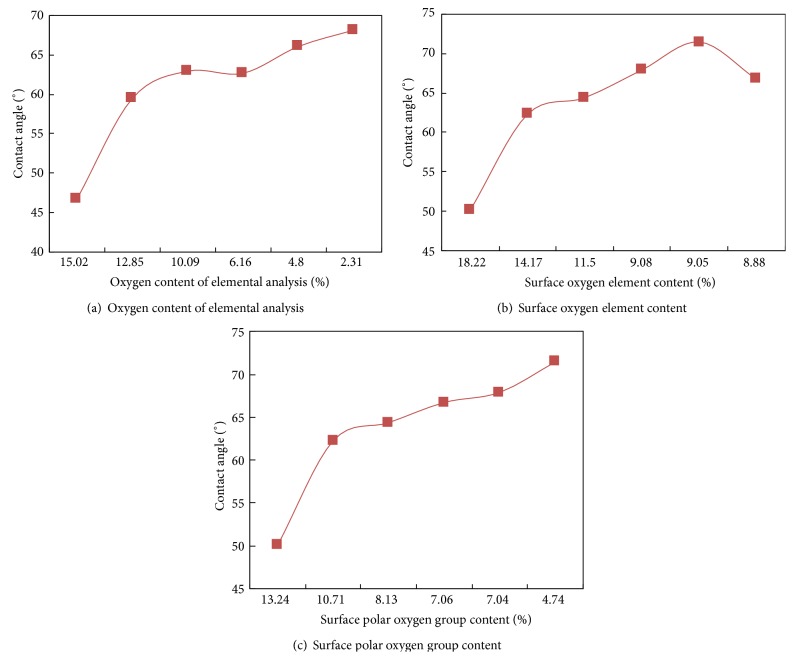
Curves of the relationship between oxygen contents and contact angles before and after deashing.

The curves of contact angle versus oxygen content (before and after being deashed) are recorded in Figure 3.

From Figure 3(a), it can be seen that, before the deashing process, various coal samples show distinctly different wettability, which is influenced by the content of oxygen. As the oxygen content declines, the contact angle gradually rises up. Among all the coal dust samples, the oxygen contents of Beizao lignite and Daliuta long flame coal are both higher than 14% and the contact angles are lower than 59.5° prior to deashing process.

It is evident from Figures 3(b) and 3(c) that, followed by a decrease of surface oxygen and oxygen-containing polar groups, the contact angle shows a steady increase. However, the negative correlation between contact angle and oxygen-containing polar groups is more obvious than the correlation between contact angle and surface oxygen. The polar oxygen group contents of both Beizao lignite and Daliuta long flame coal are higher than 10.5%, and the contact angles after being deashed are both lower than 62.5°. Due to the elimination of inorganic oxygen in the deashing process, the contact angle of all the coal samples is only influenced by polar groups and it becomes larger after the deashing process, indicating lower wettability.

It is worth noting that the surface oxygen content of Xinjulong fat coal is 8.88%. With the lowest oxygen content among all six coal samples, Xinjulong fat coal is expected to have the largest contact angle. In fact, its contact angle is measured to be merely 66.74°, lower than that of Wugou coking coal and Yangquan anthracite. The latter two have higher surface oxygen content of 9.08% and 9.05% and smaller contact angle of 67.92°and 71.47°, respectively. The reason is mainly due to the fact that Xinjulong fat coal has higher content of oxygen-containing polar group (7.06%) compared to the latter two (7.04% and 4.74%). Therefore, the content of oxygen-containing polar group can serve as a more reasonable indicator for coal dust wettability.

Beizao lignite shows lower contact angle than the other five coals both before and after deashing process. As seen in Figure 3, the contact angle has a sharp rise within the coal ranks of Beizao lignite and Daliuta coal. From Daliuta long flame coal to Yangquan anthracite, the slope of contact angle curve distinctly diminishes. The contact angle goes up with the decrease of oxygen (group) content, but the growth rate is much lower at higher metamorphic degree. For instance, the contact angle of Daliuta long flame coal after being deashed is 62.29°, which is 12.16° larger than Beizao lignite. Compared to Yangquan anthracite, however, the contact angle of Daliuta long flame coal is only increased by 9.18°, even though the metamorphic degree increases from Daliuta coal to Yangquan anthracite.

## 5. Conclusions

1. The results of elemental analysis and XPS experiments indicate that, with increasing metamorphic degree, the carbon content in coal samples increases but the oxygen content declines. However, the moisture, ash, and volatile content show no clear relevance with metamorphic degree.
2. As the metamorphic degree rises, the O/C ratio on coal surface decreases. Compared to the elemental analysis result, the O/C ratio of all the coals is higher and shows an increasing trend. This trend is more obvious among those highly metamorphic coals such as coking coal and hard coal.
3. The oxygen-containing functional groups in coal can be classified into three types: carbon-oxygen single bond (ether (C–O–C) and hydroxyl (C–OH)), carbonyl group (C=O), and carboxyl group (COO). Carboxyl group and hydroxyl group are the greatest promoter to surface wettability. As the metamorphic degree gets higher, the content of surface carboxyl and hydroxyl tends to reduce, leading to an uneven distribution of oxygen-containing polar group on the surface of different coals. The content of polar group falls from 13.24% to 4.74% as the metamorphic degree increases. The ether group, however, seems to be irregularly distributed versus the change of metamorphic degree.
4. Before the coals are deashed, the contact angle gradually rises as the oxygen content declines. After being deashed, the contact angle is still negatively related to the content of surface oxygen and polar groups. However, due to the elimination of inorganic oxygen, the contact angle of all the coals is higher than the sample before deashing process. The content of oxygen-containing polar group is a more reasonable indicator on coal dust wettability. Lignite is found to have the smallest contact angle among all the coal samples both before and after deashing process. The surface wettability is not significantly influenced by metamorphic degree for all the coal samples except for Beizao lignite.

## Figures and Tables

**Table 1:** Proximate analysis and elemental analysis of coal samples of different types.

Coal samples	Proximate analysis/%				Elemental analysis/%				
	M_ad_	A_ad_	V_ad_	FC_ad_	C_daf_	O_daf_	H_daf_	N_daf_	S_daf_
Beizao lignite	17.16	10.93	41.27	30.64	72.88	15.02	8.66	2.36	1.08
Daliuta long flame coal	10.31	6.22	52.26	31.21	80.21	12.85	4.27	0.86	1.81
Yushuwan gas coal	5.49	2.95	37.41	54.15	83.89	10.09	4.96	0.82	0.24
Xinjulong fat coal	1.55	5.6	30.56	62.29	86.14	6.16	5.32	1.79	0.59
Wugou coking coal	1.86	7.59	10.8	79.75	87.34	4.8	5.71	1.56	0.59
Yangquan anthracite	1.7	3.96	8.02	86.32	91.79	2.31	3.81	1.43	0.66

**Table 2:** Data of XPS full spectrum scan of coal samples with different metamorphic grades.

Coal samples	Atomic concentration of elements/%				(O/C) ratio/%	
	C 1s	O 1s	N 1s	S 2p	Elemental analysis	XPS analysis
Beizao lignite	80.34	18.22	1.44	/	20.61	22.68
Daliuta long flame coal	84.37	14.17	0.94	0.52	16.02	16.80
Yushuwan gas coal	87.68	11.5	0.82	/	12.03	13.12
Xinjulong fat coal	89.11	8.88	1.72	0.29	7.15	9.97
Wugou coking coal	88.41	9.08	1.83	0.68	5.50	10.27
Yangquan anthracite	89.18	9.05	1.34	0.43	2.52	10.15

**Table 3:** Ownership and content of organic carbon of different coal samples.

Coal samples	Relative concentration ratio of different forms of organic C/%			
	COO	C=O	C–O	C–H, C–C
Beizao lignite	9.22	3.85	20.37	66.56
Daliuta long flame coal	8.16	2.15	15.77	73.92
Yushuwan gas coal	4.85	3.26	10.18	81.71
Xinjulong fat coal	4.21	1.22	8.43	86.14
Wugou coking coal	5.34	4.17	7.09	83.4
Yangquan anthracite	3.05	2.88	10.92	83.15

**Table 4:** Contents of superficial oxygen-containing functional groups of different coal samples.

Coal samples	Molar content of oxygen-containing functional groups *W*_mol_/%				
	COO	C=O	C–OH	C–O–C	Polar oxygen groups (C–OH, COO)
Beizao lignite	7.41	3.09	5.83	5.27	13.24
Daliuta long flame coal	6.88	1.81	3.83	4.74	10.71
Yushuwan gas coal	4.25	2.86	3.88	2.53	8.13
Xinjulong fat coal	4.72	3.69	2.34	1.96	7.06
Wugou coking coal	3.75	2.59	3.29	2.11	7.04
Yangquan anthracite	2.72	2.57	2.02	3.86	4.74

**Table 5:** Contact angles between coal sample and distilled water before and after deashing.

Coal samples	Beizao lignite	Daliuta long flame coal	Yushuwan gas coal	Xinjulong fat coal	Wugou coking coal	Yangquan anthracite
Contact angle before deashing/°	46.70	59.47	62.93	62.66	66.06	68.14
Contact angle after deashing/°	50.13	62.29	64.39	66.74	67.92	71.47
